# Evaluation of antioxidant activity and total phenol in different varieties of *Lantana camara* leaves

**DOI:** 10.1186/1756-0500-7-560

**Published:** 2014-08-22

**Authors:** Sanjiv Kumar, Rajat Sandhir, Sudarshan Ojha

**Affiliations:** Department of Biochemistry, BMS Block, Panjab University, Chandigarh, 160014 India

**Keywords:** *Lantana camara*, Verbenaceae, Antioxidant activity, Total phenolic content, DPPH, ABTS

## Abstract

**Background:**

Phytochemicals like carotenoids, tocopherols, ascorbates and phenols present in the plants are strong antioxidants and have an important role in the health care system. There is growing interest in correlating the phytochemical constituents of a plant with its pharmacological activity. Therefore, the present study investigates the content of total phenolics, flavonoids and the antioxidant activity of four different varieties of *Lantana camara* L. (Verbenaceae) leaves by using *in vitro* antioxidant models.

**Methods:**

The leaves of Chandigarh purple variety (CPV), Palampur red variety (PRV), Chandigarh yellow turning pink variety (YTPV) and Chandigarh yellow variety (CYV) *Lantana camara* were collected and the total phenolic, flavonoid content, antioxidant and free radical scavenging activities were determined in their methanolic extracts.

**Results:**

The phenolic content was found to be highest in the CYV extract (232.99 ± 15.97 mg GAE/ g extract). The content of the flavonoids are in the order of YTPV, PRV, CPV and CYV. The IC_50_ values for the 2, 2-diphenyl-1-picrylhydrazyl (DPPH) radical scavenging test were in the order of CYV (33.30 ± 2.39) < PRV (40.32 ± 2.94) < YTPV (475.33 ± 5.20) < CPV (927.16 ± 2.88 μg/mL). The highest total antioxidant capacity was observed in CYV (222.20 ± 5.05 mg AAE/ g). The Ferric ion reducing antioxidant potential (FRAP) value of the extracts were in the order of CYV > PRV > YTPV > CPV. The IC_50_ values of 2, 2'-azino-bis-(3-ethylbenzothiazoline-6-sulfonate) (ABTS) scavenging assay for CYV, PRV, YTPV, CPV were 18.25 ± 0.19, 18.24 ± 1.82, 50.43 ± 9.49, 52.84 ± 1.82 μg/mL respectively. PRV extract showed the maximum in vitro lipid peroxidation inhibition effect with an IC_50_ value of 68.50 μg/mL which is even stronger as compared to the standard Rutin (79.69 μg/mL). The extracts showed a strong correlation between the phenolic content and their antioxidant activities. The highest correlation (r = 0.998, R^2^ = 0.997) was found between total phenolic content and ABTS scavenging assay.

**Conclusion:**

Among the four varieties investigated, CYV and PRV extracts showed strong antioxidant activities and may be used as a potential source of natural antioxidant against free radical associated diseases.

## Background

Free radicals are fundamental to many biochemical processes and represent an essential part of aerobic life and metabolism. Reactive oxygen species (ROS) mediated oxidative damage to macromolecules namely lipids, proteins and DNA have been implicated in the pathogenicity of major diseases such as cancer, rheumatoid arthritis, post ischemic reperfusion injury, degeneration process of aging, myocardial infarction, cardiovascular disease etc. [1]. Antioxidants function through protection mechanisms at several levels within cells in human body by inhibiting the formation of free radical species, intercepting radical-chained reactions, converting existing free radicals into less harmful molecules and repairing oxidative damage. Several synthetic antioxidants, such as butylated hydroxyanisol (BHA), butylated hydroxytoluene (BHT) and tert-butylhydroquinone (TBHQ) are commercially available but some adverse effects have been observed with their continuous use [2].

Fundamental biochemical processes of plants are considered to be the primary metabolic processes that generally follow similar mechanism in the cells of all plants and are necessary for each plant to survive and to reproduce. However, the plants produce a large number of compounds called secondary metabolites with diverse physiological and biochemical activities. Secondary metabolites and their derivatives show significant pharmacological activities such as being hepatoprotective, diuretic, anti allergic, anticancer effects and antioxidant [3–5]. Phytochemicals like carotenoids, tocopherols and tocotrienols, ascorbates and phenols are strong antioxidants and have carved out an important role in the health care system. There is renewed interest in finding out new natural antioxidants from living system for application in food, pharmaceutical and cosmetics.

*Lantana camara* L. is regarded both as a notorious weed and a popular ornamental garden plant and has found various uses in folk medicine in many parts of the world [6]. Different parts of *Lantana* plant are considered antiseptic, antispasmodic, carminative and diaphoretic [7]. Anti-inflammatory, antipyretic and analgesic properties of extracts of *L. camara* leaves have also been reported [8]. Verbascoside isolated from the leaves of *L. camara* exhibited inhibition of protein kinase C and antitumor activity [9].

The different varieties of *L. camara* have been reported to contain different types and levels of lantadenes and other phytochemicals [10], and may contain different bioactive constituents among these varieties of this *Lantana* species.

The present work aimed (i) to conduct a quantitative analyses of the antioxidant activity, total phenolic content and total flavonoids in methanolic extracts of different varieties of *Lantana camara* leaves (ii) to correlate the total phenolic content with the antioxidant activities as such comparative antioxidant study of these different varieties of *L. camara* has not been reported.

## Methods

### Chemicals and reagents

Gallic acid, 2,2-diphenyl-1-picrylhydrazyl (DPPH), 2, 4, 6-tripyridyl-s-triazine (TPTZ) and 2,2′-azino-bis-(3-ethylbenzothiazoline-6-sulfonate) (ABTS) were purchased from Sigma-Aldrich. Ascorbic acid, Folin-Ciocalteu’s reagent, sodium carbonate and silica gel F_254_ TLC plates were from Merck (Mumbai, India). All the chemicals used including solvents were of analytical grade.

### Collection of plant material

The leaves of different varieties were collected from different areas in and around Panjab University campus, Chandigarh and red variety was obtained from I.V.R.I. Palampur surroundings. The samples were authenticated and voucher specimens (Voucher specimen No. 20319 I-IV) deposited at the Herbarium, Department of Botany, Panjab University, Chandigarh. The leaves were washed, dried and grinded to make powders and then they were labelled and stored for further use. The different varieties of *Lantana camara* collected were Chandigarh purple variety (CPV), Palampur red variety (PRV), Chandigarh yellow turning pink variety (YTPV) and Chandigarh yellow variety (CYV).

### Methanolic extract preparation

Lantana leaf powders were extracted with methanol (10% w/v) for 24 hours and filtered through four layers of muslin cloth and then through Whatman No. 1 filter paper. The extracts were then subjected to rotary vaporizer (Equitron, Roteva – 8763 RV) to evaporate methanol. The semi-solid residue obtained was blackish brown in color, henceforth called *Lantana camara* methanolic extract (LCME). The percentage yield (w/w) obtained for different varieties were CPV (14.22%), PRV (16.82%), YTPV (14.46%) and CYV (15.72%).

### Estimation of total phenolic content

The total phenols were determined by method of McDonald et al., [11] with slight modifications. 10 mg of gallic acid monohydrate was dissolved in 100 mL of methanol to give a concentration of 100 μg/mL and used as the standard. Different aliquots of 0.1 to 1.0 mL from the stock solution were taken in 10 graduated tubes. To each tube 2.5 mL of 1:1 mixture of Folin- Ciocalteu reagent and distilled water and 2 mL of 7.5% sodium carbonate were added. The mixture was allowed to stand for 30 minutes and the volume was made with water to get a concentration ranging from 1–10 μg/mL. The absorbance of the resulting solutions was measured at 765 nm against reagent blank. A standard calibration curve was prepared by plotting absorbance against concentration and it was found to be linear over this concentration range. 10 mg of extract was dissolved in 10 mL of methanol to get 1 mg/mL solution The concentration of total phenol in the test sample was determined from the calibration graph. The total phenol content in the extract was expressed in terms of gallic acid equivalent (mg GAE /g extract).

### Estimation of total flavonoid content

Aluminium chloride colorimetric technique was used for flavonoids estimation [12]. 10 mg of rutin was dissolved in 10 mL of methanol to get 1000 μg/mL solution and was used as standard. Aliquots ranging from 0.01 to 0.08 mL from the above stock solution were taken in different tubes. To each tube 1.5 mL of methanol, 0.1 mL of 10% aluminium chloride, 0.1 mL of 1 M potassium acetate and 2.8 mL of distilled water was added. The reaction mixture was kept at room temperature for 30 min. The absorbance of the resulting solutions was measured at 415 nm against reagent blank. The calibration curve was prepared by plotting absorbance against concentration and it was found to be linear over this concentration range. 10 mg of extracts were dissolved in 10 mL of methanol to get 1 mg/mL solutions respectively. The concentration of total flavonoid in the test sample was determined from the calibration curve. The total flavonoid content in the extract was expressed as rutin equivalent (mg RE/g extract).

### Thin layer chromatography analysis of antioxidant activity

DPPH (2,2-Diphenyl-1-picrylhydrazyl) assay [13] was used as a rapid thin layer chromatography screening method to evaluate the antioxidant activity of the methanolic extracts due to free radical scavenging. DPPH is a purple coloured stable free radical, which on reduction gives yellow coloured diphenyl picryl hydrazine compound. 2.5 μL of different methanolic extracts (1 mg/mL) were loaded on silica gel F_254_ TLC plates (Merck, Germany) which were sprayed with 0.05% DPPH solution in methanol and examined at 30 minutes after spraying. Any antioxidant compound is seen as a yellow spot on a purple background. Vitamin C and gallic acid were used as positive controls.

### DPPH radical scavenging activity

The DPPH free radical scavenging activity of the extract, based on the scavenging of the stable 2, 2-diphenyl-1-picrylhydrazyl (DPPH) free radical was determined [13]. It is a discoloration assay, which is evaluated by the addition of the antioxidant to a DPPH solution in methanol and the ability to scavenge the stable free radical of DPPH was measured in the absorbance at 517 nm.

The solution of DPPH in methanol (200 μM) was freshly prepared. Different concentrations of extracts were added to methanolic solution of DPPH. After 30 min at room temperature in dark, the absorbance was recorded at 517 nm. IC_50_ value denotes the concentration of sample, which is required to scavenge 50% of DPPH free radicals. Radical scavenging activity was calculated by the following formula


IC_50_ value was determined from the plotted graph of scavenging activity against the different concentrations of extracts, which is defined as the total antioxidant necessary to decrease the initial DPPH radical concentration by 50%. The measurements were triplicated and their scavenging effects were calculated based on the percentage of DPPH scavenged.

### Evaluation of total antioxidant capacity

The assay is based on the reduction of Mo (VI) to Mo (V) by the extract and subsequent formation of a green phosphate/Mo (V) complex at acidic pH [14]. 0.3 ml extract was mixed with 3 ml of reagent solution (0.6 M sulphuric acid, 28 mM sodium phosphate and 4 mM ammonium molybdate). The tubes containing the reaction solutions were incubated at 95°C for 90 min. Then the absorbance of the solution was measured at 695 nm using a spectrophotometer against blank after cooling to room temperature. Methanol (0.3 ml) in place of extract was used as blank. The antioxidant activity was expressed as ascorbic acid equivalent (mg AAE/g extract) which served as a positive control.

### Ferric ion reducing antioxidant potential (FRAP) assay

A modified method of Benzie & Strain [15] was adopted for the FRAP assay. The stock solutions prepared were 300 mM acetate buffer (3.1 g CH_3_COONa and 16 mL CH_3_COOH), pH 3.6, 10 mM TPTZ (2, 4, 6-tripyridyl-s-triazine) solution in 40 mM HCl, and 20 mM FeCl_3_ solution. The fresh working solution was prepared by mixing 25 mL acetate buffer, 2.5 mL TPTZ and 2.5 mL FeCl_3_ solution. The temperature of the solution was raised to 37°C before using. Different concentrations of plant extracts (100 μL) were allowed to react with FRAP solution (2900 μL) for 30 min in the dark condition. Readings of the coloured product (ferrous tripyridyltriazine complex) were taken at 593 nm. The standard curve was linear between 200 to 1000 μM FeSO_4._ Results are expressed in μM Fe (II)/ g extract and compared with BHT as standard.

### ABTS radical scavenging assay

The method of Re et al., [16] with slight modifications was adopted for ABTS (2,2′-azino-bis-(3-ethylbenzothiazoline-6-sulfonate) assay. Briefly, a stock solution of ABTS radical cation was prepared by dissolving ABTS (7 mM) with potassium persulfate (K_2_S_2_O_8_, 2.45 mM). The mixture was left to stand in the dark at room temperature for 16 h (the time required for formation of the radical) before use. For the evaluation of ABTS radical scavenging activity, the working solution was prepared by the previous solution and diluting it in ethanol to obtain the absorbance of 0.700 ± 0.02 at 734 nm. The solvent extracts (0.1 mL) at different concentrations were mixed with the ABTS working solution (1.9 mL) and the reaction mixture was allowed to stand at 30°C for 6 min, then the absorbance was measured by using a UV-visible spectrophotometer at 734 nm, at which point the antioxidants present in the extracts began to inhibit the radical, producing a reduction in absorbance, with a quantitative relationship between the reduction and the concentration of antioxidants present in the tested sample. The radical scavenging activities of the extracts were compared with that of BHT and percentage inhibition is calculated by equation:


### *In vitro*lipid peroxidation inhibition assay

Rat liver was processed to get 10% homogenate in cold phosphate buffered saline, pH 7.4 using polytron homogenizer and filtered to get a clear homogenate. The degree of lipid peroxidation was assayed by estimating the TBARS by using the method of Ohkawa et al., [17] with slight modifications. Different concentrations of the extracts (20–100 μg/ mL) were added to liver homogenate. Lipid peroxidation was initiated by adding 50 μL of 15 mM ferrous sulphate solution to 0.2 mL of homogenate. After 1 h, 1 mL of 15% TCA followed by 1 mL of 0.67% TBA in 50% acetic acid was added to the tubes and heated in boiling water bath for 30 min. The tubes were centrifuged and the intensity of the pink coloured complex was measured at 532 nm. The control was prepared without fractions and rutin was used as the standard. The experimental procedure was approved by the Institutional animal ethics committee, Panjab University, Chandigarh. Experiments involving animals were conducted in accordance with the guidelines for the use of laboratory animals.

### Statistical analysis

Assays were performed in triplicate and results are shown as mean ± standard deviation. Linear regression analysis was used to calculate the IC_50_ values. Pearson’s correlation coefficient was calculated using Microsoft excel 2007. Statistical significance was determined among various treatments with one way ANOVA test using SPSS 16.0 for Windows. A statistical significance of p < 0.05 was considered to be significant.

## Results and discussion

Phenolic compounds are considered to be the most important antioxidants and are widely distributed among various plant species. These phenols play important roles in plants such as protection against herbivores and pathogens, cementing material joining phenolic polymers to cell wall polysaccharides [18], regulation of cell growth and cell division [19]. The phenolic content expressed as gallic acid equivalent per gram (GAE /g) in the CYV extract was found to be highest (232.99 ± 15.97 mg GAE/g) followed by PRV (225.15 ± 12.52 mg GAE/g), YTPV (70.91 ± 4.57 mg GAE/g) and CPV (55.57 ± 2.82 mg GAE/g). Phenolic compounds exhibit their antioxidant activity by several mechanisms such as donating hydrogen atoms to free radicals, scavenging other reactive species such as OH^•^, NO_2_^•^, N_2_O_3,_ ONOOH and HOCl. Some phenolics, mostly the di and polyphenols, can react with O_2_^•‾^ or by binding transition metal ions (especially iron and copper), often resulting in forms poorly active in promoting free radical reactions and hence can also interfere with the uptake of metals from the diet [20, 21]. Phenolic acids such as caffeic acid and rosmarinic acid were reported to be among the abundant compounds in the methanolic extracts of aerial tissues of *L. camara* [22]. Vamanu and Nita showed that rosmarinic acid, a major phenolic compound, in the methanolic extracts of wild edible *Boletus edulis* mushroom is an effective antioxidant [23].

Flavonoids are water soluble polyphenolic compounds which are extremely common and wide spread in the plant kingdom as their glycosides. The contents of total flavonoid were measured by aluminium chloride method and are expressed in terms of rutin equivalent (RE) as 16.14 ± 0.21 mg RE/ g for CYV, 18.17 ± 0.92 mg RE/g for CPV, 24.60 ± 2.25 mg RE/ g for PRV, 25.22 ± 2.59 mg RE/g for YTPV (Table 1). The flavonoids act through scavenging or chelating process [24, 25]. Ghisalberti mentioned six flavonoids, such as 3-methoxy-, 3, 7 dimethoxy- and 3, 7, 4′-trimethoxyquercetin, pectolinarigénin 7-O-β-D-glucoside, hispidulin and a camaraside glycoside in the leaves and stems of *L. camara* [7].

**Table 1:** **Total phenolic content (mg GAE /g extract) and flavonoid content (mg RE /g extract)**

Sample	Total phenolic content (mg GAE / g) ± SD	Flavonoid content (mg RE/ g) ± SD
CPV	55.57 ± 2.82^b^	18.17 **±** 0.92^b^
PRV	225.15 ± 12.52^a^	24.60 **±** 2.25^a^
YTPV	70.91 ± 4.57^b^	25.22 **±** 2.59^a^
CYV	232.99 ± 15.97^a^	16.14 **±** 0.21^c^

Results are expressed as mean ± SD (n = 3). Gallic acid equivalent (GAE) and rutin equivalent (R.E.).

Values in the column followed by a different letter superscript (a-c) are significantly different (p < 0.05) and values having same superscript are not statistically significant.

**Figure 1:**
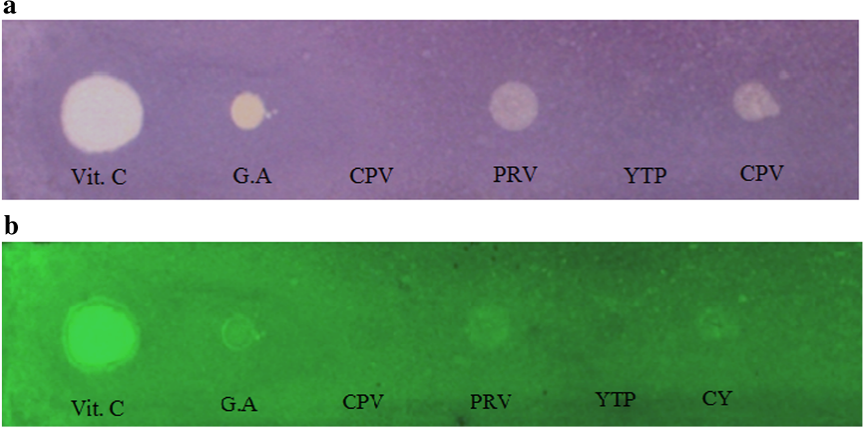
**Antioxidant activity by qualitative DPPH test on F**_**254**_ **TLC plate (Merck). a)** in visible light **b)** in U.V. light at 254 nm. Vit. C (Vitamin C), G.A. (Gallic acid), CPV (Chandigarh Purple), PRV (Palampur red), YTPV (Yellow turning pink), CYV (Chandigarh yellow).

The result of the qualitative DPPH test on TLC plate (F_254_) is shown in Figure 1. The DPPH scavenging effects are expressed as yellow zones of the various extracts on the purple background as compared to positive controls ascorbic acid (Vitamin C) and gallic acid and blank TLC plate as negative control.

Table 2 shows the IC_50_ values of leaf extracts for DPPH free radical scavenging activity for CYV (33.30 ± 2.39 μg/ mL), PRV (40.32 ± 2.94 μg/ mL), YTPV (475.33 ± 5.20 μg/ mL), CPV (927.16 ± 2.88 μg/ mL). The lower IC_50_ value indicates higher antioxidant capacity and hence CYV extract showed significantly higher radical scavenging activity as compared to PRV, YTPV and CPV. The radical scavenging activity is in accordance with the levels of phenolic content in the extracts respectively. Free radical scavenging activity of extracts of black chokeberry and blueberry has been reported to be correlated with its total phenolics content [26].

**Table 2:** **Effect of methanolic extracts of** ***Lantana camara*** **on different antioxidant models**

Sample	DPPH radical scavenging activity IC _50_Value (μg/mL)	Total antioxidant capacity (mg AAE /g) ± SD	FRAP value (mM Fe (II)/g) ± SD
CPV	927.16 ± 2.88^e^	105.70 **±** 0.80^d^	5.60 **±** 0.25^d^
PRV	40.32 ± 2.94^c^	210.30 **±** 0.41^b^	236.30 **±** 6.11^c^
YTPV	475.33 ± 5.20^d^	155.40 **±** 2.72^c^	17.70 **±** 2.95^d^
CYV	33.30 ± 2.39^b^	222.20 **±** 5.05^a^	339.00 **±** 11.13^b^
Vitamin C	10.73 ± 0.09^a^	-	-
BHT	-	-	550.00 **±** 7.54^a^

Results are expressed as mean ± SD (n = 3). Ascorbic acid equivalent (AAE). Values in the column followed by a different letter superscript (a-e) are significantly different (p < 0.05) and values having same superscript are not statistically significant. -, not determined.

Total antioxidant capacity is a better way of depiction of combined effect of phenolics, flavonoids and other reducing compounds in the plant extracts and is expressed in terms of ascorbic acid equivalents (AAE). The phosphomolybdenum method is based on the reduction of Mo (VI) to Mo (V) by the action of antioxidant compounds and the formation of a green phosphate - Mo (V) complex with a maximal absorption at 695 nm. A significantly higher total antioxidant capacity was observed in CYV (222.20 ± 5.05 mg AAE/ g) followed by PRV (210.30 ± 0.41 mg AAE/ g), YTPV (155.40 ± 2.72 mg AAE/ g) and CPV (105.70 ± 0.80 mg AAE/ g) (Table 2). The total antioxidant capacity values follow the same order as that of phenolic content in the extracts respectively.

The ferric ion reducing antioxidant potential (FRAP) of different extracts were estimated from their ability to reduce TPTZ-Fe (III) to TPTZ-Fe (II). The FRAP value of the extracts is in the order of BHT (550.00 ± 7.54 mM Fe (II)/g) > CYV (339.00 ± 11.13 mM Fe (II)/g) > PRV (236.30 ± 6.11 mM Fe (II)/g), YTPV (17.70 ± 2.95 mM Fe (II)/g) followed by CPV (5.60 ± 0.25 mM Fe (II)/g) (Table 2). The differences in the values are found to be significant and are in order with their phenolic content.

**Figure 2:**
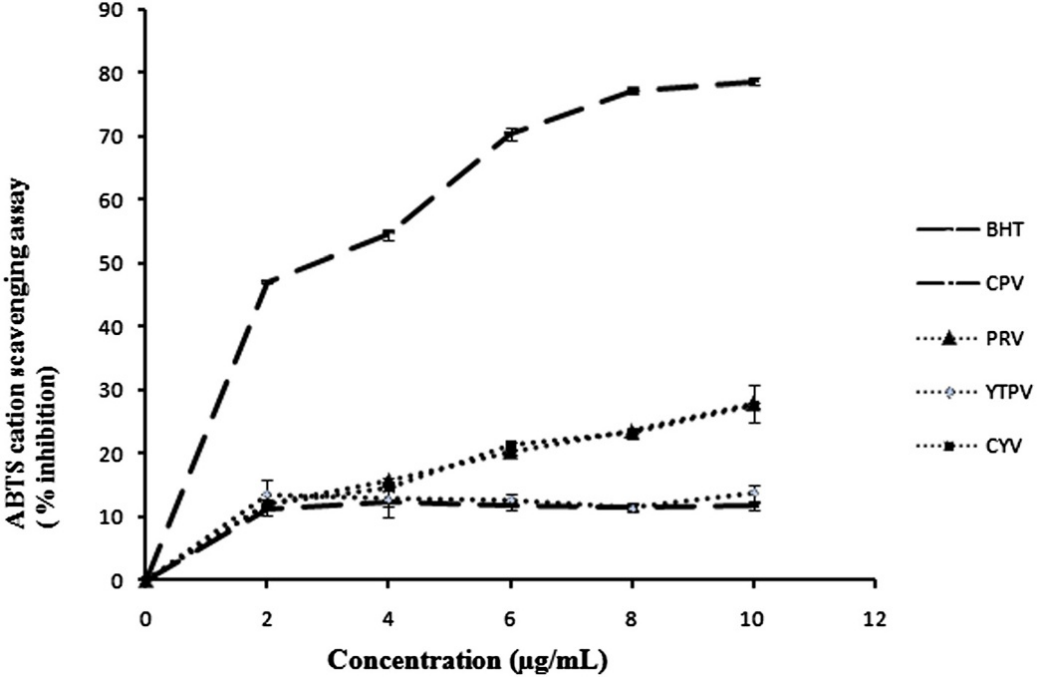
**ABTS radical cation scavenging activity.**

Protonated radical scavenging is an important attribute of antioxidants. ABTS, a protonated radical, has characteristic absorbance maxima at 734 nm which decreases with the scavenging of the proton radicals [27]. The extracts were fast and effective scavengers of the ABTS cation radical (Figure 2). The IC_50_ values for PRV (18.24 ± 1.82 μg/mL), CYV (18.25 ± 0.19 μg/mL), YTPV (50.43 ± 9.49 μg/mL) and CPV (52.84 ± 1.82 μg/mL) are given in Table 3.

**Table 3:** **Effect of methanolic extract of CPV, PRV, YTPV and CYV compared with BHT as standard on ABTS radical scavenging assay**

Conc. (μg/mL)	[IC _50_values are mean ± SD of 3 replicates] Inhibition (%)				
	BHT	CPV	PRV	YTPV	CYV
2	47.15 ± 0.32	11.13 ± 0.45	12.05 ± 0.31	13.60 ± 2.32	12.10 ± 0.73
4	54.61 ± 0.85	12.29 ± 0.59	15.74 ± 0.58	12.82 ± 2.84	14.67 ± 0.59
6	70.55 ± 0.95	11.66 ± 0.39	20.26 ± 0.38	12.68 ± 0.91	21.28 ± 0.52
8	77.30 ± 0.51	11.56 ± 0.17	23.47 ± 0.25	11.51 ± 0.52	23.17 ± 0.77
10	78.71 ± 0.53	11.66 ± 0.25	27.78 ± 2.92	13.79 ± 1.38	27.50 ± 0.44
**IC**_**50**_ **(μg/mL)**	**4.34 ± 0.03** ^**a**^	**52.84 ± 1.82** ^**c**^	**18.24 ± 1.17** ^**b**^	**50.43 ± 9.49** ^**c**^	**18.25 ± 0.19** ^**b**^

Values in the row followed by a different letter superscript (a-c) are significantly different (p < 0.05) and values having same superscript are not statistically significant.

**Figure 3:**
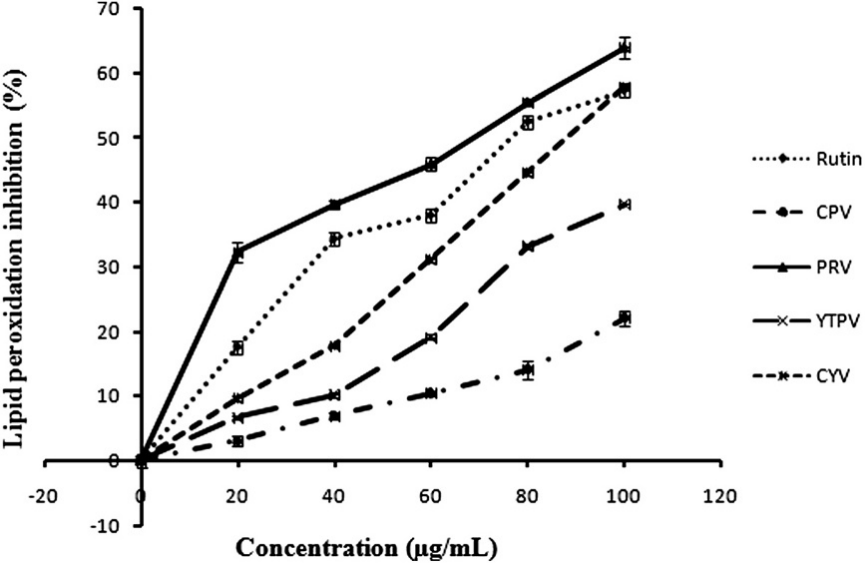
***In vitro*** **lipid peroxidation inhibition activity.**

Lipid peroxidation is the oxidative degradation of polyunsaturated fatty acids and involves the formation of lipid radicals leading to membrane damage. Free radicals induce lipid peroxidation in polyunsaturated lipid rich areas like brain and liver [28] and can also damage DNA, proteins and other biological molecules. Initiation of lipid peroxidation by ferrous sulphate takes place either through ferryl-perferryl complex [29] or through hydroxyl radical by Fenton reaction [30] depending upon the reaction conditions. The extracts showed the *in vitro* lipid peroxidation inhibition effects in a concentration dependent manner and the results are expressed in terms of percentage inhibition (Figure 3) and IC_50_ values for the same are calculated from the regression equation respectively (Table 4). PRV extract showed the significantly maximum inhibitory effect with lowest IC_50_ value of 68.51 ± 0.48 μg/ mL, CYV (89.77 ± 0.72 μg/ mL), YTPV (127.59 ± 3.03 μg/ mL), CPV (242.78 ± 6.96 μg/ mL) as compared to the standard rutin which was showing the inhibitory effect with an IC_50_ value of 79.68 ± 0.66 μg/ mL. Here, PRV showed higher inhibitory effect than rutin (1 mg/ mL) used as a standard. Lipid peroxidation inhibiting activity of *Lantana* extracts may be due to the compounds such as rosmarinic acid, caffeic acid etc. Caffeic acid (3,4-dihydroxycinnamic acid) has been shown to prevent the lipid peroxidation of food induced by the free radicals and also in pathological conditions, such as cancer and ageing [31]. Aqueous extract of *Lantana camara* has been reported to inhibit pBR322 plasmid DNA damage induced by free radicals [32] indicating the presence of compounds with strong antioxidant properties.

**Table 4:** **Effect of methanolic extract of CPV, PRV, YTPV and CYV compared with Rutin as standard on** ***in vitro*** **lipid peroxidation inhibition assay in rat liver**

Conc. (μg/mL)	[IC _50_values are mean ± SD of 3 replicates] Inhibition (%)				
	Rutin	CPV	PRV	YTPV	CYV
20	17.62 ± 1.05	3.09 ± 0.67	32.24 ± 1.56	6.76 ± 0.27	9.66 ± 0.45
40	34.33 ± 1.00	6.99 ± 0.51	39.67 ± 0.59	10.19 ± 0.34	17.8 ± 0.26
60	38.00 ± 1.00	10.43 ± 0.38	45.91 ± 1.00	19.18 ± 0.44	31.14 ± 0.36
80	52.47 ± 0.91	14.14 ± 1.40	55.48 ± 0.50	33.23 ± 0.35	44.62 ± 0.45
100	57.24 ± 1.03	22.14 ± 1.17	63.95 ± 1.69	39.76 ± 0.09	57.86 ± 0.35
**IC**_**50**_ **(μg/mL)**	**79.68 ± 0.66** ^**b**^	**242.78 ± 6.96** ^**e**^	**68.51 ± 0.48** ^**a**^	**127.58 ± 3.03** ^**d**^	**89.77 ± 0.72** ^**c**^

Values in the row followed by a different letter superscript (a-e) are significantly different (p < 0.05) and values having same superscript are not statistically significant.

**Figure 4:**
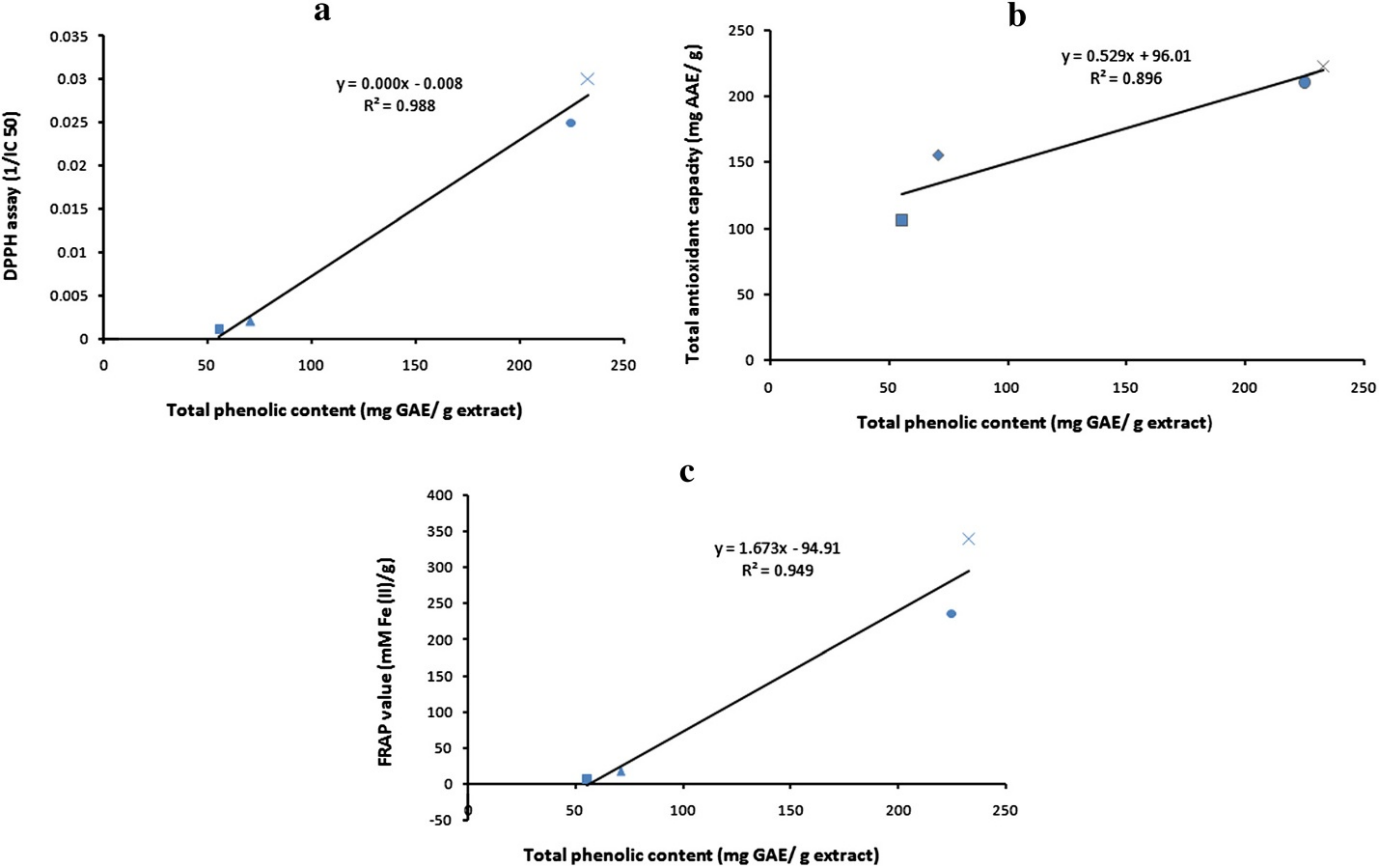
**Correlation between total phenolic content and (a) DPPH radical scavenging activity of the extracts.** Correlation coefficient, r = 0.994 and coefficient of determination, R^2^ = 0.988. **(b)** Total antioxidant capacity of the extracts. Correlation coefficient, r = 0.946 and coefficient of determination, R^2^ = 0.896. **(c)** Ferric ion reducing antioxidant potential (FRAP) of the extracts. Correlation coefficient, r = 0.974 and coefficient of determination, R^2^ = 0.949.

The correlation of total phenolic content with DPPH, FRAP and ABTS scavenging activities are shown in Figure 4(a), (b) and (c) respectively. The Pearson’s correlation coefficient (r) and coefficient of determination (R^2^) was highest (r = 0.998, R^2^ = 0.997) between total phenolic content and ABTS activity than that of total phenolic content and DPPH activity (r = 0.994, R^2^ = 0.988) followed by total phenolic content and FRAP activity (r = 0.974, R^2^ = 0.949).

**Figure 5:**
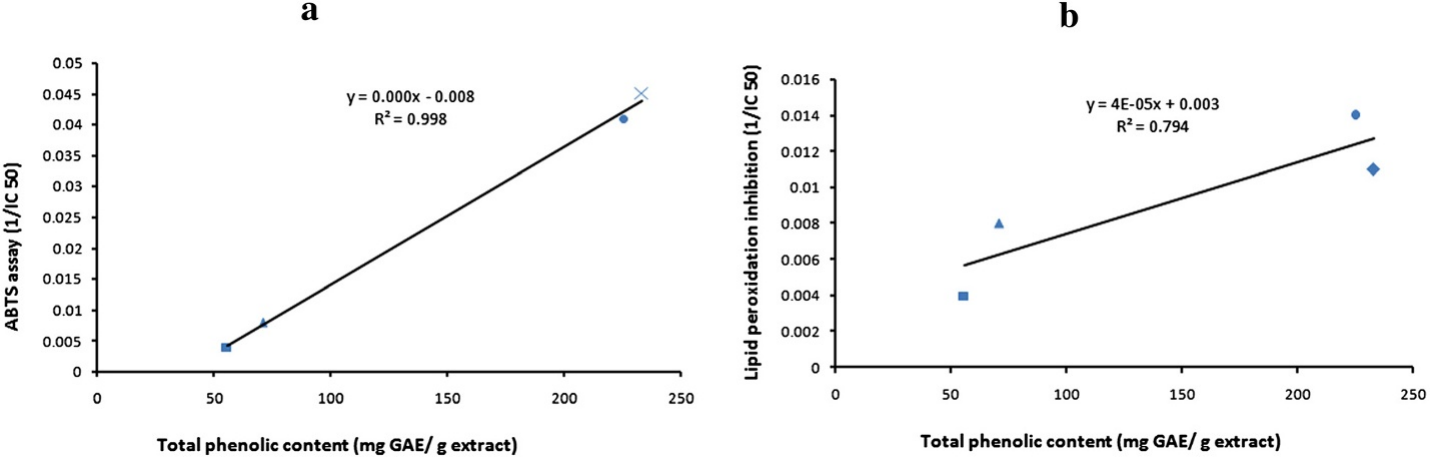
**Correlation between total phenolic content and (a) ABTS scavenging activity of the extracts.** Correlation coefficient, r = 0.998 and coefficient of determination, R^2^ = 0.997 **(b)** Lipid peroxidation inhibition activity of the extracts. Correlation coefficient, r = 0.891and coefficient of determination, R^2^ = 0.794.

The correlation between total phenolic content with total antioxidant capacity and *in vitro* lipid peroxidation inhibition activity is shown in Figure 5 (a) and (b) respectively. A correlation (r = 0.946, R^2^ = 0.833) was observed in Figure 5 (a) and a relatively lower correlation (r = 0.891, R^2^ = 0.794) in case of Figure 5 (b). Reports have suggested that there is a correlation between the total phenolic content and antioxidant activity of plant extracts [33]. However, no correlation is found between the flavonoid content and the antioxidant activities of the extracts in the present study.

The high correlations confirm the role of phenolic compounds as the main contributor to the antioxidant activities of the *Lantana camara* leaf extracts. The types and quantities of phenolic compounds might contribute to the varying antioxidant activity of extracts from different varieties. Some phenolic compounds such as salicylic acid, gentisic acid, β-resorcylic acid, coumarin, ferulic acid and 6-Methyl coumarin were identified in *L. camara* extract [34].

## Conclusion

On the basis of the results obtained in the present study, it is concluded that the methanolic leaf extracts of four different varieties of *Lantana camara* which contains large amount of phenolic compounds, exhibit high antioxidant, free radical scavenging and *in vitro* lipid peroxidation inhibition activities. A high correlation is found between the total phenolic content and the antioxidant activities using different *in vitro* antioxidant models in this study. These *in vitro* assays indicate that these four different varieties has a varying antioxidant activity, and thereby indicate that among them CYV and PRV varieties are good source of antioxidants, which might be useful in preventing the progress of various oxidative conditions. Hence, more queries will be addressed in future studies targeting these varieties to explore the potential and isolation of bioactive compounds responsible for such activities and as chemo preventive and therapeutic agents.

## Abbreviations

AAE: Ascorbic acid equivalent
RE: Rutin equivalent
GAE: Gallic acid equivalent
TLC: Thin layer chromatography
DPPH: 2,2-diphenyl-1-picrylhydrazyl
FRAP: Ferric ion reducing antioxidant potential
ABTS: 2,2′-azino-bis-(3-ethylbenzothiazoline-6-sulfonate)
ROS: Reactive oxygen species
CPV: Chandigarh purple variety
PRV: Palampur red variety
YTPV: Yellow turning pink variety
CYV: Chandigarh yellow variety.
